# Performance of Field-Collected *Spodoptera frugiperda* (Lepidoptera: Noctuidae) Strains Exposed to Different Transgenic and Refuge Maize Hybrids in Argentina

**DOI:** 10.1093/jisesa/iez110

**Published:** 2019-12-16

**Authors:** María G Murúa, Martín A Vera, Andrew Michel, Augusto S Casmuz, Julio Fatoretto, Gerardo Gastaminza

**Affiliations:** 1 Instituto de Tecnología Agroindustrial del Noroeste Argentino, Estación Experimental Agroindustrial Obispo Colombres, Consejo Nacional de Investigaciones Científicas y Técnicas (ITANOA-EEAOC-CONICET), Las Talitas, Tucumán, Argentina; 2 Estación Experimental Agroindustrial Obispo Colombres (EEAOC), Las Talitas, Tucumán, Argentina); 3 Department of Entomology, Ohio Agricultural Research and Development Center, The Ohio State University, Wooster, OH; 4 Syngenta Crop Protection, São Paulo, Brazil

**Keywords:** fall armyworm, Cry1F, resistant strain, larval duration, asynchronous emergence

## Abstract

*Spodoptera frugiperda* (J. E. Smith) is one of the major pests of maize in Argentina. The main tool for its control is the use of genetically modified maize hybrids expressing *Bacillus thuringiensis* (Bt) insecticidal proteins. Maize growers in Argentina initially controlled this pest with Bt maize that expressed a single Bt protein (Cry1Ab or Cry1F). Currently it is necessary to plant maize cultivars that produce two Bt proteins to achieve the satisfactory control. Recently, Cry1F field-evolved resistant populations of this species were confirmed in Argentina. The objective of this study was to evaluate the performance of *S. frugiperda* field-collected strains on different Bt and non-Bt maize hybrids. Strains were collected from non-Bt maize (T1), Agrisure TDMax (T2), Agrisure Viptera (T3), Agrisure Viptera 3110 (T4), Genuity VT Triple Pro (T5), and Power Core (T6). Three experiments were performed to 1) determine the survivorship and reproduction of field-collected larvae (F_0_) from Bt maize hybrids, 2) evaluate Cry1F resistance using an F_1_ screen, and 3) assess the performance of F_1_ strains on different maize hybrids. In the F_0_, the survivorship from larva to adult ranged from 0 to 63%. We obtained adults from only the T1, T2, T5, and T6 strains with no significant differences in the reproductive parameters. Continuously rearing F_1_ larvae on their collected hosts affected larval duration, which was significantly shorter for a known-laboratory Bt-susceptible strain than the field-collected strains. Our results support the existence of Cry1F-resistance alleles in *S. frugiperda* field populations in Argentina.

*Spodoptera frugiperda* (J. E. Smith), the fall armyworm, is one of the major agricultural pests in the Western Hemisphere, infesting maize (*Zea mays* L.), sorghum (*Sorghum* spp.), turf grasses, and a number of other crops (Luginbill 1928, Casmuz et al. 2010). In maize grown in northern Argentina, fall armyworm is the most important insect pest, causing yield losses that fluctuate from 17 to 72% (Perdiguero et al. 1967, Willink et al. 1993ab). Currently, the most widely used tool for fall armyworm control in Argentina is the use of the genetically modified maize hybrids expressing *Bacillus thuringiensis* (Bt) insecticidal proteins (Blanco et al. 2010, Huang et al. 2014). Herculex I (TC1507), which expresses the Cry1F protein, was launched during the 2005–2006 seasons in Argentina and has been widely adopted due to its high level of efficacy against fall armyworm. However, fall armyworm has evolved resistance to the Cry1F maize in the country and threatens the durability of this trait for control (Chandrasena et al. 2017).

Fall armyworm resistance to Cry1F was first reported in Puerto Rico (Storer et al. 2010). Later reports were documented in Florida, Brazil, and North Carolina (Huang et al. 2014, Farias et al. 2014, 2016). In Argentina, the introduction of late season plantings, limited refuge compliance and prevalence of multiple pest generations in the north have increased both the exposure to Cry1F and the selection pressure for resistance to Cry1F maize (Trumper 2014, Argenbio 2019). Monitoring studies of Bt hybrids expressing Cry1F performed in the most important maize growth regions in Argentina from 2009 to 2015 confirmed resistance of fall armyworm field populations to the Cry1F maize (Chandrasena et al 2017). Similarly, field studies demonstrated that fall armyworm damaged several maize hybrids expressing Cry1F in different Argentinian regions (Flores and Balbi 2014, Trumper 2014, Balbi and Flores 2015). Such a rapid increase in resistance is a serious concern for the continued use of Bt traits against fall armyworm. In most cases, the inheritance of Cry1F resistance in fall armyworm populations was characterized as autosomal, incompletely recessive and monogenic in Puerto Rico, Brazil, and Argentina (Storer et al. 2010, Velez et al. 2013, Farias et al. 2014, Chandrasena et al. 2017).

Resistance to Bt proteins can be delayed using insect resistant management (IRM) strategies (Tabashnik et al. 2008). The success of these strategies includes several assumptions which include a high dose of the Bt protein, a low initial resistant allele frequency, random mating between resistant and susceptible insects, and the presence an abundance of a non-Bt refuge. The purpose of the refuge is to reduce selection pressure by the Bt protein and provide susceptible insects to mate with resistant insects (Tabashnik et al. 2008). However, the frequency of susceptible insects in the refuge depends on several factors, including pest bionomics, genetic mode and stability of resistance, and the relative performance of susceptible and resistant insects. Comparing the performance of susceptible and resistant insects on Bt maize hybrids can help understand fitness costs and improve IRM (Wu et al. 2002).

Although there are a lot of research that examined fitness costs of Bt resistance in fall armyworm (Jakka et al. 2014, Vélez et al. 2014, Dangal and Huang 2015, Horikoshi et al. 2016), it is important to know how Cry1F-resistant fall armyworm performs on other Bt maize hybrids that have the same or different toxins for managing the Cry1F resistance in fall armyworm. Greenhouse trials by Niu et al. (2014) evaluated larval survivorship and leaf injury of Cry1F-susceptible (SS), resistant (RR), and heterozygous (RS) genotypes of fall armyworm on whole plants of non-Bt and Bt hybrids. They found that both the larval survivorship and leaf injury rates of the RR larvae on Cry1F corn plants were not significantly different from those observed on non-Bt corn hybrids. They demonstrated that pyramided Bt corn containing Cry2 and or Vip3A can be used for managing the Cry1F resistance in fall armyworm. Yang et al. (2016) evaluated larvae of SS, RR, and RS genotypes of fall armyworm on pyramided and single-gene Bt cotton. They found that all genotypes were susceptible to the pyramided cotton and the single-gene cotton containing Cry2Ae, whereas the pyramided cotton containing Cry1Ac/Cry1F was effective against SS and RS, but not for RR. These results showed that the Cry1F-maize–selected fall armyworm can cause cross-crop resistance to other Bt crops expressing similar insecticidal proteins. Muraro et al. (2019) evaluated the survival of fall armyworm strains on Bt and non-Bt maize in laboratory and field conditions and its susceptibility to insecticides. They found that RR larvae reared on Bt and non-Bt maize showed a similar susceptibility to spinetoram and chlorfenapyr. In the field trials, no differences in fall armyworm survival were detected between strains when the commercial dose of two insecticides was applied in Bt and non-Bt maize. All these information are relevant since Cry1F-resistant individuals may be frequently found in refuges instead of purely susceptible insects. If there are differences in the performance between susceptible and Cry1F-resistant larvae in the refuge (including developmental delays), it may affect random mating between moths emerging from the Bt crop and refuge areas, and significantly decrease the efficacy of the refuge (Gryspeirt and Gregoire 2012, Jakka et al. 2014). The objectives of this study were to 1) determine the performance of fall armyworm collected from various Bt maize hybrids in the field after continuous rearing on the same collected hybrid and 2) evaluate the performance of fall armyworm resistance to Cry1F when continuously reared on non-Bt and Bt maize hybrids grown in the field. By evaluating the biological parameters of fall armyworm strains over multiple generations, we found developmental delays in the field-collected populations, relatively to a known Bt-susceptible laboratory strain, which could affect the success of Bt maize IRM in fall armyworm.

## Materials and Methods

### Insect Collections

Six fall armyworm strains were collected on 18 January 2017 from six different commercial maize hybrids: non-Bt maize (T1) (Hybrid: SYN 840 TG PLUS-Syngenta), Agrisure TDMax (Cry1Ab) (T2) (Hybrid: NK 907 TD/TC-Syngenta), Agrisure Viptera (Vip3Aa20) (T3) (Hybrid: NK 900 Viptera 3-Syngenta), Agrisure Viptera 3110 (Vip3Aa20 and Cry1Ab) (T4) (Hybrid: SYN 139 Viptera-Syngenta), Genuity VT Triple Pro (Cry1A.105, Cry2Ab2, and Cry3Bb1) (T5) (Hybrid: DK 7310-Monsanto), and Power Core (Cry1F, Cry1A.105, Cry2Ab2) (T6) (Hybrid: Dow 507 PW-Dow). At the time of collecting, the maize growth stage of all hybrids was V4-V5 (Ritchie and Hanway 1982). All collections were made in the same field located at La Cruz county (26°6′S, 64°9′W), Tucumán province, Argentina. Except for T3 and T4, we collected 30 larvae (instars 4–5) and placed them individually in glass tubes (12 cm high and 1.5 cm diameter) containing Bt or non-Bt maize leaves from the sampled hybrids. We only found 3 (L4 and L5) and 2 (L4) larvae in hybrids T3 and T4, respectively.

### Host Plants

We grew the same hybrids from the field (T1–T6) in the greenhouse using plastic pots (15 cm in diameter and 600 ml) filled with sterilized soil (one seed per pot). Maize seeds were planted on fertilized soil with nitrogen (45 kg/10,000 m^3^) during the planting (30%) and V4 (70%) (recommended for cultivation in Northwestern Argentina). Plants were maintained in the greenhouse under ambient lighting at approximately 33 ± 4°C, 80 ± 10% RH, and 14:10 (L:D) h. The growth stages of plants used to feed the larvae were V4 to V6 (Ritchie and Hanway 1982). In each experiment, fresh leaf tissues were excised from the greenhouse-grown plants to feed the larvae. The expression of the expected Cry proteins in the maize plants was confirmed using the qualitative ELISA Quickstix lateral flow detection strips (Envirologix, Portland, ME).

### Experiments

#### Experiment 1: Survivorship and Reproduction of Field-Collected Fall Armyworm Larvae (F_0_) From Different Bt Maize Hybrids

Field-collected larvae from different maize hybrids (T1–T6) were taken to the laboratory and placed in growth chambers under identical controlled conditions (27 ± 2°C, 70–75% RH, and a photoperiod of 14:10 [L:D] h). These insects were considered as the F_0_ generation and fed on fresh leaf tissue of its respective hybrid from where they were collected until the pupal stage. According to the methodology described by Murúa et al. (2008), larvae were placed individually in glass tubes (12 cm in height and 1.5 cm in diameter) with fresh leaf tissue which was supplemented every 2 or 3 d. As larvae pupated, pupae were sexed and placed in 100-ml plastic pots until adult emergence. We measured larval survivorship as the number of larvae reaching adult stage (single larva was a replicate). We also determined the duration of the pupal stage, pupal mass (obtained 24 h after pupation), and the adult sex ratio.

According to Murúa et al. (2008), one virgin female adult and one virgin male adult (24 h old) from the same treatment were single-pair mated (i.e., T1 × T1; T2 × T2; T5 × T5; T6 × T6) in a cylindrical polyethylene-terephthalate oviposition cage (30 cm in height and 10 cm in diameter) to determine adult longevity and reproductive parameters. We established 9, 6, 7, and 9 single-pairs mated of T1, T2, T5, and T6 treatments. For aeration, nylon mesh covered the top of cage as well as a hole on one side. The cages contained small pieces of paper on which the female laid eggs. Adults were provided a moist cotton wick containing honey and water (1:1; vol:vol), which was replaced every day. Daily mortality and oviposition were recorded from each cage until adult death. Immediately after death, females were dissected and inspected for spermatophores in their reproductive tracts to determine whether mating had occurred (Perfectti 2002, Rhainds 2010). We measured several reproductive parameters: preoviposition (the period from female emergence to first egg mass), oviposition (period from first to last eggs mass), postoviposition (period from the last eggs mass to female death), total fecundity (number of eggs laid by each female), total fertility (percentage of eggs hatching), and adult longevity (days alive).

#### Experiment 2: Evaluation of Cry1F Resistance Using an F1 Screen

Considering that field-evolved Cry1F resistance in fall armyworm occurs throughout different regions of Argentina (Chandrasena et al 2017), we evaluated if any of our fall armyworm strains also carried Cry1F resistance. F_1_ larvae from the T1, T2, T5, and T6 strains were exposed to fresh leaf tissue of Herculex maize (Hybrid: TC1507 Dow AgroSciences LLC and Pioneer Hi-Bred International), which expresses Cry1F protein (note we did not obtain enough F_0_ larvae in T3 and T4). Larvae were placed individually in glass tubes (12 cm in height and 1.5 cm in diameter) with fresh leaf tissue of Herculex maize which was supplemented every 2 or 3 d. As larvae pupated, pupae were sexed and placed in 100-ml plastic pots until adult emergence. Insects rearing was under controlled conditions (27 ± 2°C, 70–75% RH, and a photoperiod of 14:10 [L:D] h). We exposed the F_1_ larvae for one generation, reporting larval survivorship as the number of larvae reaching the adult stage. Each treatment included four replicates with each replicate consisting of approximately 40 larvae; as a control we simultaneously exposed 16 larvae of each insect strain to fresh leaf tissue of non-Bt maize (Hybrid: SYN 840 TG PLUS-Syngenta). Mortality was assessed daily and survivorship calculated at the conclusion of the experiment.

#### Experiment 3: Performance of F_1_ Fall Armyworm Strains Exposed to Different Maize Hybrids Under Controlled Conditions

The performance of F_1_ fall armyworm colonies (T1, T2, T5, and T6) was estimated with neonates reared on its respective maize hybrids [non-Bt maize (Hybrid: SYN 840 TG PLUS-Syngenta) T1, Agrisure TDMax (Hybrid: NK 907 TD/TC-Syngenta) T2, Genuity VT Triple Pro (Hybrid: DK 7310-Monsanto) T5, and Power Core (Hybrid: Dow 507 PW-Dow) T6] and compared against a Cry1F-susceptible laboratory colony (SS) feeding on non-Bt maize (Hybrid: SYN 840 TG PLUS-Syngenta). The SS colony was initiated from 1500 larvae sampled from non-Bt maize (Hybrid: SYN 840 TG PLUS-Syngenta) fields in Tafí Viejo (26°44′S, 41°13′W) and Los Pereiras (26°55′S, 64°53′W) counties (Tucumán province), in 2008. This colony was documented to be highly susceptible to Cry1F protein because it showed 99% of mortality when the larvae were feed with fresh leaf tissue of Herculex maize (unpublished data). This colony was maintained on fresh, non-Bt maize leaves (Hybrid: SYN 840 TG PLUS-Syngenta). Growth chamber conditions were identical for both susceptible and resistant strains (27 ± 2°C, 70–75% RH, and a photoperiod of 14:10 [L:D] h).

Neonates of each treatment were randomly selected from a pool of eggs laid during four different days. At the first day of eclosion, 50, 50, 80, and 140 neonates of T1, T2, T5, and T6, respectively (per cohort), were individually transferred to Petri dishes containing a fresh leaf from a hybrid of its original collection, which was supplemented every 2 or 3 d. After 2 d, the procedure was repeated for the second cohort, along with a third cohort (4 d after the first cohort) and a fourth cohort (6 d after the first cohort). The total number of larvae of each colony tested was 194, 222, 322, and 588 of T1, T2, T5, and T6, respectively. Larval mortality was recorded daily. According to Murúa et al. (2008), once larvae pupated, pupae were sexed and placed in 100-ml plastic pots until adult emergence. Then, one virgin female adult and one virgin male adult (24 h old) from the same treatment were single-pair mated in cylindrical polyethylene-terephthalate oviposition cages (30 cm in height and 10 cm in diameter). For aeration, nylon mesh covered the top of cage as well as a hole on one side. The cages contained small pieces of paper on which the female laid eggs. Adults were provided a moist cotton wick containing honey and water (1:1; vol:vol), which was replaced every day.

To generate F_1_ offspring, we set up single pair matings: 10 pairs for T1; 18 pairs for T2; 0 pairs for T5, 4 pairs for T6; and 20 pairs for SS (Table 2). Data collected from daily evaluations were used to calculate the total developmental time, measured from the first eggs mass laid to adult mortality.

Biological (duration of egg, larval and pupal stages, pupal mass, obtained 24 h after pupation, and adult sex ratio) and reproductive (presence and number of spermatophores, preoviposition, oviposition and postoviposition period duration, total fecundity, total fertility, and adult longevity) parameters were evaluated to measure the impact of continuous exposure to Bt proteins on fall armyworm performance (Table 4).

### Statistical Analysis

An initial analysis was made to test for the normality of the data using the Shapiro–Wilk’s test. Differences across treatments among egg, larval, pupal stage, pupal mass, number of spermatophores transferred, preoviposition, oviposition and postoviposition period duration, total fecundity, and total fertility and adult longevity were determined by analysis of variance (ANOVA) or Kruskal and Wallis (1952) test (*P* < 0.05) when data were not normally distributed. For each strain, we calculated corrected survival according to Henderson and Tilton (1955).

For the reproductive parameters of preoviposition, oviposition, and postoviposition periods, we included only those females that laid eggs. Total fecundity was compared among all females, including those that did not lay eggs. For total fertility, females that laid eggs but had no spermatophores were not included.

Statistical analyses were performed using Infostat version 2015p (Di Rienzo et al. 2008).

## Results

### Experiment 1: Survivorship and Reproduction of Field-Collected Fall Armyworm Larvae (F_0_) From Different Bt Maize Hybrids

The larval survivorship ranged from 0 to 63% depending on the hybrid. Notably, fall armyworm collected from T3 (*n* = 3) and T4 (*n* = 2) did not survive continuous exposure to their respective maize plants that contained Vip3Aa20. In the remaining treatments (T1, T2, T5, and T6), there was no significant difference in the duration of the pupal stage or the pupal mass (Table 1).

**Table 1.** Fall armyworm larval (F_0_) performance in the laboratory on maize hybrids of the original field collection

	Treatments					
	T1	T2	T3	T4	T5	T6
	Non Bt maize	Agrisure TDMax	Agrisure Viptera	Agrisure Viptera 3110	Genuity VT Triple Pro	Power Core
No. of collected larvae	33	33	3	2	30	33
No. of parasitized larvae	1	4	0	0	4	0
Pupa (d)* (*N*)	10.6 ± 0.3a (20)	10.2 ± 0.3a (17)	0	0	9.9 ± 0.2a (14)	10.4 ± 0.2a (21)
Pupal mass (mg)* (*N*)	200.3 ± 6.6a (20)	192.8 ± 6.9a (17)	–	–	213.4 ± 9.4a (14)	210.2 ± 6.4a (21)
No. of Female obtained	9	11	–	–	7	9
No. of Male obtained	11	6	–	–	7	12
Total survivorship (%)	60	51	0	0	46	63

d = days; *N* = sample size.

*Mean ± SE reported; total number in parentheses.

Values followed by same letters within a row for each strain are not significantly different according to ANOVA test (*P* > 0.05).

Fall armyworm larvae collected and reared on non-Bt (T1) had the longest adult survival (15.4 d); however, this difference was not significant when compared with Bt maize (T2, T5, and T6; Table 2). Of the total number of females obtained in T5 (seven), only three females contained spermatophores (Table 2), but this result was also not significantly different from other treatments. In fact, we did not observe any significant differences among the reproductive parameters of fall armyworm adults, even for the F_0_ larvae collected from Bt maize that were able to complete development under continuous Bt exposure (e.g., T2, T5, and T6).

**Table 2.** Reproduction parameters of F_0_ fall armyworm adults exposed to their respective maize hybrids

Reproductive parameters	Crosses ♀ × ♂							
	T1x T1	*N*	T2 × T2	*N*	T5 × T5	*N*	T6 × T6	*N*
Female adult Longevity** (d)	15.4 ± 1.4a	9	13.2 ± 1.7a	6	13.6 ± 2.3a	7	12.8 ± 1.7a	9
Male adult Longevity** (d)	9.3 ± 1.2a	9	10.8 ± 0.9a	6	13 ± 1.6a	7	12.3 ± 0.9a	9
Spermatophores per female*	1.2 ± 0.4a	9	0.8 ± 0.2a	6	1 ± 0.6a	7	1.1 ± 0.3a	9
Mated female	6		5		3		8	
Preoviposition period* (d)	6.4 ± 1.2a	5	4.4 ± 0.7a	5	4.7 ± 1.7a	3	4.9 ± 0.8a	8
Oviposition period** (d)	7.4 ± 1.4a	5	6.4 ± 1.2a	5	6.3 ± 1.2a	3	6.2 ± 0.8a	8
Postoviposition period* (d)	0.2 ± 0.2a	5	0.8 ± 0.5a	5	1 ± 0.6a	3	0.2 ± 0.2a	8
Fecundity**	1136.7 ± 138.6a	4	1600.2 ± 180.9a	5	1615.7 ± 339.2a	3	1580.2 ± 184.1a	8
Fertility*	99.3 ± 0.7a	4	100 ± 0a	5	100 ± 0a	3	99.6 ± 0.4a	8

T1 = non-Bt maize; T2 = Agrisure TDMax; T3 = Agrisure Viptera; T4 = Agrisure Viptera 3110; T5 = Genuity VT Triple Pro; T6 = Power Core.

*N* = number of single pair matings; d = days; mean ± S.E. reported.

*Values followed by same letters within a row are not significantly different according to Kruskal–Wallis– test (*P* > 0.05).

**Values followed by same letters within a column for each strain are not significantly different according to ANOVA test (*P* > 0.05).

### Experiment 2: Estimation of Cry1F Resistance Allele Using an F1 Screen

We determined whether any of our fall armyworm strains had resistance alleles to Cry1F. We evaluated a total of 165, 164, 110, and 166 larvae (F_1_) tested from T1, T2, T5, and T6, respectively. Indeed, we measured high survival on fresh leaves of Cry1F with T1 (79 larvae, 47.8% survival) and T2 (78 larvae, 47.6% survival) strains. Larval survival from T5 (0 larvae) and T6 (4 larvae, 2.4% survival) was extremely low (Table 3). All larval survivors of Cry1F exposure reached the adult stage in T1, T2, and T6 strains.

**Table 3.** Mortality (M) (%) and survivorship (S) (%) of fall armyworm larvae from Tucumán province exposed to fresh tissue of Cry1F (Herculex) maize

Rep.	T1			T2			T5			T6		
	Control^*a*^	M	S	Control	M	S	Control	M	S	Control	M	S
1	12	56 (23)	44 (18)	16	51 (21)	49 (20)	16	100 (22)	0	16	97.5 (40)	2.5 (1)
2	16	59 (24)	41 (17)	15	49 (20)	51 (21)	14	100 (31)	0	16	95.1 (39)	4.9 (2)
3	16	51 (21)	49 (20)	16	56 (23)	44 (18)	16	100 (22)	0	16	100 (42)	0
4	16	43 (18)	57 (24)	16	54 (22)	46 (19)	16	100 (35)	0	15	97.6 (41)	2.4 (1)
Average	100	52.2 (86)	47.7 (79)	100	52.5 (86)	47.5 (78)	100	100 (110)	0	100	97.5 (162)	2.4 (4)
Corrected survival		47.9			47.6			0			2.4	

(*N*) = number of individuals evaluated. T1 = non–Bt maize; T2 = Agrisure TDMax; T5 = Genuity VT Triple Pro; T6 = Power Core.

^*a*^The number of fall armyworm larvae exposed to non–Bt corn; average in these columns are average survival.

### Experiment 3: Development and Reproduction of Field-Collected Fall Armyworm Strains (F_1_) When Fed on Its Respective Maize Hybrid of Field Collection

In T5, none of the 31 larvae survived continuous exposure and were therefore not included for most of the performance comparisons. Several parameters were significantly different among the other strains including the egg stage period (*H* = 19.48; *P* = 0.0001), larval stage period (*H* = 173.05; *P* = 0.0001), pupal stage period (*H* = 38.93; *P* = 0.0001), pupal mass (*F =* 207.24; df = 3, 213; *P* = 0.0001), and male longevity (*H* = 27.02; *P* = 0.0001). The larval stage duration was significantly shorter in the SS strain (18.33 d) compared with any of the resistance strains (>34 d). Among the T1, T2, and T6 strains, the duration of the larval stage ranged from 34.1 to 40.4 d, with the T2 strain having a significantly shorter development time than T6 strain. In addition, the pupal mass of the SS strain (224.6 g) was significantly greater than any of the field-collected strains (ranging from 109.1 to 135.4 g). Adults from T1, T2, and T6 had significantly less longevity, but only the males were significantly different from the susceptible strain. Combined, our data show that all fall armyworm strains significantly differed in longevity, taking approximately 57, 53, 60, and 47 d, for T1, T2, T6, and SS, respectively, to complete a single generation (from egg to adult mortality) under laboratory conditions. For most of the larval and pupal characteristics, the SS strains on non-Bt leaf tissue had the most optimum phenotypes (Table 4).

**Table 4.** Development and reproductive parameters of field-collected (F1) and susceptible (SS) fall armyworm strains exposed to different maize hybrids

Life cycle stages	T1	*n*	T2	*n*	T5	*n*	T6	*n*	SS	*n*
Egg*	3.1 ± 0.1b	56	2.9 ± 0.1ab	80	2.9 ± 0.1ab	31	3 ± 0.1b	49	2.7 ± 0.1a	253
Larva*	35.1 ± 0.4bc	41	34.1 ± 0.3b	70	–	–	40.4 ± 1.1c	14	18.3 ± 0.3a	98
Pupa*	8.78 ± 0.2b	40	7.7 ± 0.1a	52	–	–	8.8 ± 0.2bc	11	9.3 ± 0.2c	65
Total from egg to Adult emerged**	46.9 ± 0.6b	40	44.5 ± 0.5b	52	–	–	51.6 ± 1.1c	11	31.4 ± 0.5a	65
Pupal mass**	135.4 ± 3.6b	40	109.1 ± 4.5a	52	–	–	121.8 ± 8.4ab	11	224.6 ± 3.1c	114
Female adult longevity*	9.7 ± 1.4a	10	11.4 ± 1.4a	18	–	–	10.7 ± 3.1a	4	12.7 ± 1.1a	20
Male adult longevity*	11.4 ± 1.4a	10	7.7 ± 0.5a	18	–	–	6.7 ± 1.3a	4	16.6 ± 1.3b	20
Sex ratio ♀:♂	0.7:1	40	0.89:1	51	––	–	1.8:1	11	1.5:1	79
Spermatophores per female*	1.2 ± 0.2a	8	1 ± 0a	9	–	–	1.2 ± 0.2a	4	1.4 ± 0.1a	19
Preoviposition period*	5.3 ± 0.7a	7	5.4 ± 1.1a	9	–	–	4.2 ± 0.6a	4	3.5 ± 0.4a	18
Oviposition period**	2.1 ± 0.5a	1	2.2 ± 0.4a	9	–	–	4 ± 0.9ab	4	7.4 ± 0.9b	18
Postoviposition period*	1.3 ± 0.5a	7	0.9 ± 0.4a	9	–	–	2.7 ± 2.4a	4	2.2 ± 0.5a	18
Total fecundity**	312.7 ± 89.7a	7	261.1 ± 42.9a	9	–	–	317.7 ± 87.7a	4	1061.2 ± 144.8b	18
Total fertility*	88.7 ± 4.9a	7	83.1 ± 10.4a	9	–	–	73.8 ± 16.3a	4	72.5 ± 8.5a	18
Development time (egg to adult died)	56.9 ± 0.9bc	20	53.3 ± 1.1ab	36	–	–	60.4 ± 3.1c	4	47.7 ± 1.1a	40

T1 = non-Bt maize; T2 = Agrisure TDMax; T5 = Genuity VT Triple Pro; T6 = Power Core; SS = susceptible strain (fed on non-Bt maize).

Duration in days (mean ± SE) of egg, larval, and pupal stages, pupal mass (g), female and male longevity (days), sex ratio (F:M), number of spermatophores per female, duration of preoviposition, oviposition and postoviposition periods (d), total fecundity, total fertility (%), and development time (d).

*Values followed by same letters within a row are not significantly different according to Kruskal–Wallis test (*P* > 0.05).

**Values followed by same letters within a column for each strain are not significantly different according to ANOVA test (*P* > 0.05).

**Fig. 1.**
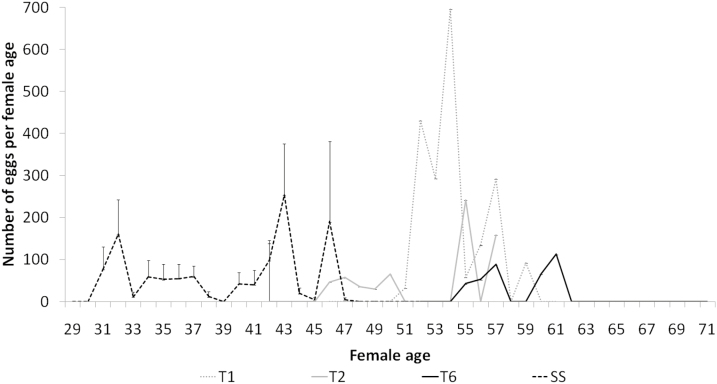
Oviposition patterns of three fall armyworm field-collected and susceptible strains at different female ages. A total of 8 females were used for T1 (non-Bt maize), 9 females for T2 (Agrisure TDMax), 4 females for T6 (Power Core), and 19 females for SS (susceptible strain).

Both the oviposition period (*F* = 8.6; df = 3, 34; *P* = 0.0002) and total fecundity (*F* = 9.16; df = 3, 34; *P* = 0.0001) were significantly different among fall armyworm strains. For both parameters, the SS strain had a significantly longer oviposition period and a significantly greater number of laid eggs (Table 4). Over time, the peak times of egg laying differed among strains, with the SS having a substantially earlier peak (43 d) than any of the field-collected strains (ranging from 54 to 61 d, Fig. 1). Nonetheless, fall armyworm continuously reared on T2 and T6 for the F_0_ and an F1 screen were able to complete development on Bt maize with high fertility.

## Discussion

Fall armyworm resistance to Bt crops presents a significant challenge, especially for crops in tropical and subtropical regions where the larva is considered a key pest. IRM strategies to extend the durability of Bt crops must be a priority. Among these practices, a structured refuge (i.e., block of 10% non-Bt maize planted with 90% of Bt maize) associated with a high dose is considered the most efficient IRM strategy. However, with Cry1F resistance in high frequency in South America (Table 3; Farias et al. 2014, 2016, Chandrasena et al. 2017), the performance and emergence of fall armyworm from the refuge may shift and affect the frequencies of resistance to Bt traits other than Cry1F. In this study, we found significant differences in the performance of different fall armyworm strains resistant to Cry1F when reared on Bt hybrids.

In our first experiment, we collected fall armyworm from several Bt hybrids that did not contain the Vip3Aa20 protein, and surprisingly, about half of the larvae collected from these Bt hybrids were able to complete development and produce an F_1_ screen (Tables 1 and 2), despite continuous exposure to Bt. In addition, these collections did not differ from those collected from non-Bt hybrids (e.g., T1) in any phenotypic characteristics (Tables 1 and 2). In the F1 screen, we split our strains to rear them on Cry1F or continue the Bt selection as in the field F_0_ collections. For the T1 and T2 strains, survival on Cry1F was relatively high, almost reaching 50% corrected survival (Table 3). It is important to mention that all larval survivors of Cry1F exposure reached the adult stage. This survival value was higher than that reported by Muraro et al. (2019). They found that Cry1F-resistant fall armyworm showed 76 and 32% of larval and adults survival on Cry1F (Herculex) maize, respectively. This survival clearly supports the existence of resistance alleles in field populations, as this survival was detected even for fall armyworm from T1 (non-Bt). As observed in other cases (Niu et al. 2014, Bernardi et al. 2015), Cry1F resistance results in cross-resistance to Cry1Ab (T2) but not to Vip3Aa20 (T3). On the other hand, the result with T5 and T6 clearly demonstrates that the Cry1F-resistant fall armyworm are susceptible to Cry2Ab, as this is the only toxin that would be active against Cry1F-resistant in the T5 and T6 hybrids. This may be due to lack of cross-resistance with this trait. These results are consistent with those reported by Niu et al. (2014). They did not fount live larvae of the Cry1F-resistant fall armyworm on plants of four pyramided Bt maize, including two hybrids with the same proteins of T5 and T6. This is likely due to the lack of cross-resistance between Cry1A presented in T5 and T6 (Hernández Rodríguez et al. 2013, Bernardi et al. 2015). Thus, the resistance phenotype is similar to the one described in Puerto Rico, Florida, and Brazil (Velez et al. 2013, Huang et al. 2014, Farias et al. 2014, 2016).

After confirming the Cry1F resistance, we continued exposure to Bt hybrids for the F1 screen for the fall armyworm strains of T1, T2, T5, and T6. Except for T5 (Table 4), all strains were able to complete a second generation of continuous exposure to their respective Bt hybrid. However, these field-collected fall armyworm strains significantly differed in many of the biological and reproductive traits compared with a Cry1F susceptible strain. Larval longevity was perhaps the most affected parameter, especially for T6, whose larvae took more than twice as long, to pupate than the SS strain (Table 4). This observation was also reported by Jakka et al. (2014). The T1, T2, and T6 strains also had negative effects on reproduction parameters. Oviposition period and total fecundity were both significantly greater in the susceptible strain. Our strains were less fecund than other fall armyworm– resistant strains (Vélez et al. 2014, Horikoshi et al. 2016, Muraro et al 2019).

When the development times of all life stages are combined, the susceptible strain had a significantly shorter time than the T1, T2, and T6 strains. In our study, the main factor was likely the shorter development time of the larvae (18.33 d). The delayed larval development caused an asynchronous emergence of the adults from the susceptible and resistant strains. According to our data, susceptible adults emerged 13–23 d earlier than resistant strains of T2 and T6. Therefore, if this situation would develop in the field, in natural conditions, the susceptible adults would not likely encounter nor mate with the emerging adults from resistant strains. This behavior will affect the random mating between moths emerging from a Bt crop and refuge areas (Gryspeirt and Gregoire 2012, Jakka et al. 2014).

Our results support the existence of resistance alleles of Cry1F in fall armyworm field populations in Argentina and showed that Cry1F Cry protein may have reduced efficacy in this country. On the other hand, our results showed that the single-gene Cry1Ab maize product (T2) was not effective against fall armyworm. These results proved that the Cry1F-resistant fall armyworm are susceptible to Cry2Ab, toxin present in T5 and T6 hybrids. This may be due to lack of cross-resistance with this trait. These results would suggest that these pyramided Bt maize technologies can be used for managing the Cry1F resistance in fall armyworm. These results have important implications to resistance management.
